# Revisiting Animal Reservoirs of Hantavirus and Other Zoonotic Viruses Associated with Cruise Travel: Impact and Prevention

**DOI:** 10.3390/ani16182966

**Published:** 2026-09-21

**Authors:** D. Katterine Bonilla-Aldana, Jorge L. Bonilla-Aldana, Ivan Camilo Sanchez-Rojas, Lysien I. Zambrano, Alfonso J. Rodríguez-Morales

**Affiliations:** 1 College of Medicine, Korea University, Seoul 02841, Republic of Korea; dbonilla@korea.ac.kr; 2 Grupo de Virologia, Universidad El Bosque, Bogotá 111321, Colombia; jlbonilla@unbosque.edu.co; 3 Grupo de Investigación en Genética, Biodiversidad y Manejo de Ecosistemas (GEBIOME), Universidad de Caldas, Manizales 170004, Colombia; ivan.sanchez@itp.edu.co; 4 Instituto de Investigaciones One Health, Universidad Tecnológica Centroamericana (UNITEC), Tegucigalpa 11101, Honduras; lysienzambrano@unitec.edu; 5 Faculty of Health Sciences, Universidad Científica del Sur, Lima 15074, Peru; 6 Grupo de Investigación Biomedicina, Faculty of Medicine, Fundación Universitaria Autónoma de las Américas-Institución Universitaria Visión de las Américas, Pereira 660003, Colombia

**Keywords:** orthohantavirus, viral zoonoses, international cruises, one health, travel medicine

## Abstract

The sustained growth of international maritime tourism and the expansion of the cruise industry have increased the importance of emerging zoonotic diseases as a significant challenge to global public health. Cruise ships constitute complex epidemiological settings characterized by high population density, rapid international mobility, and constant interaction between humans, animals, and coastal environments, factors that favor the introduction and spread of emerging infectious agents. Potential zoonotic concerns include orthohantaviruses and other zoonotic viruses, such as dengue, chikungunya, Zika, yellow fever, and avian influenza, which are associated with animal reservoirs and vectors in ports, tourist destinations, and tropical ecosystems. This review examines the ecology of animal reservoirs of orthohantaviruses and other zoonotic pathogens relevant to maritime tourism, including peri-urban rodents, bats, migratory birds, and other mammals. It discusses how climate change, coastal urbanization, and environmental change affect viral transmission dynamics. It highlights epidemiological risks linked to international cruises, particularly exposure during ecotourism excursions, pathogen introduction, and challenges for maritime surveillance. The review also addresses travel medicine, biosecurity, and infection control, emphasizing a One Health approach. Strengthening integrated surveillance and prevention systems is essential to ensure safer, more sustainable, and resilient maritime tourism in the 21st century.

## Simple Summary

Cruise ships connect passengers and crews with ports, coastal environments, wildlife, and animal reservoirs across multiple regions. This review examines how rodents, bats, migratory birds, non-human primates, and other animals may contribute to the circulation of orthohantaviruses and other zoonotic viruses relevant to maritime tourism. It also discusses how climate change, urbanization, ecotourism, and international mobility may increase exposure risks. Effective prevention requires rodent and vector control, environmental sanitation, traveler education, early detection, and coordinated surveillance. A One Health approach that integrates human, animal, and environmental health is essential for safer, more resilient cruise travel.

## 1. Introduction

The sustained expansion of international maritime tourism over the past few decades has transformed cruise ships into one of the most dynamic sectors of the global tourism industry [1]. Before the COVID-19 pandemic, millions of passengers traveled annually on vessels connecting multiple continents, ports, and ecosystems in relatively short periods. In fact, global cruise passenger numbers reached approximately 29.7 million in 2019, reflecting the scale and international reach of the cruise industry before the pandemic [2]. While the pandemic highlighted the vulnerability of cruise ships to respiratory and highly transmissible infectious diseases, it also underscored the need to expand surveillance to other emerging infectious agents, particularly those of zoonotic origin, in line with One Health approaches to pandemic preparedness [3]. In this context, cruise ships represent complex environments where high population density, international mobility, environmental exposure, and indirect contact with animal reservoirs and vectors converge, creating conditions conducive to the introduction and dissemination of emerging and re-emerging viruses [4,5].

Among the viral zoonoses of increasing importance are orthohantaviruses, members of the genus Orthohantavirus within the family Hantaviridae. The human-pathogenic rodent-borne viruses discussed in this review include Andes virus (ANDV; species *Orthohantavirus andesense*) and Seoul virus (SEOV), both members of the genus *Orthohantavirus* within the family *Hantaviridae* and maintained primarily in specific wild or synanthropic rodent reservoirs [6]. These infections can produce severe clinical pictures, including orthohantavirus cardiopulmonary syndrome and hemorrhagic fever with renal syndrome, both associated with high morbidity and mortality [7].

Although orthohantaviruses have traditionally been linked to rural areas and agricultural activities, urban expansion, environmental alteration, and increasing human mobility have created new opportunities for interaction between humans and rodent reservoirs, extending potential exposure to peri-urban environments, transport hubs, storage facilities, and tourism-related settings [8,9]. Particularly relevant is the role of the Seoul virus, associated with rats of the genus *Rattus*, widely distributed in port cities and commercial vessels around the world [10].

The close ecological association between urban rats and maritime infrastructure makes Seoul virus particularly relevant in international shipping and cruise-related environments [11]. The historical role of maritime trade in the global dissemination of *Rattus* species may also contribute to the worldwide circulation of this orthohantavirus in port ecosystems [12].

In addition to orthohantaviruses, mosquito-borne viruses such as dengue, chikungunya, Zika, and yellow fever represent important travel-associated risks for cruise passengers and crews. For these infections, exposure is expected to occur predominantly in endemic port destinations and during shore excursions or other terrestrial activities rather than onboard the vessel itself [13].

Likewise, emerging viruses associated with bats, migratory birds, and other mammals have become increasingly important due to increased ecotourism and excursions in jungle, coastal, or island areas [14]. Interactions among susceptible travelers, biodiverse ecosystems, and environmental changes favor increasingly complex transmission scenarios, especially amid climate change, coastal urbanization, and the expansion of maritime routes into new ecological areas [15,16,17].

From a One Health perspective, cruises are dynamic interfaces where human, animal, and environmental health converge [18]. Epidemiological surveillance in these scenarios requires multidisciplinary approaches that integrate travelers’ medicine, maritime public health, reservoir ecology, vector control, and environmental monitoring [19]. However, despite the growing relevance of viral zoonoses in international tourism, the role of animal reservoirs in the epidemiology of cruise ship-associated infections remains poorly integrated conceptually and operationally [20].

This review analyzes the ecology and diversity of animal reservoirs involved in orthohantaviruses and other zoonotic viruses relevant to cruise travel across the complete travel continuum. For clarity, we distinguish four principal evidence settings: (i) zoonotic pathogens or reservoirs documented aboard cruise ships; (ii) pathogens or reservoirs documented in seaports, commercial vessels, or other maritime infrastructure; (iii) infections associated with shore excursions, destination exposure, or acquisition before embarkation; and (iv) theoretical or extrapolated risks derived from terrestrial, wildlife, or ecological evidence. These categories are not considered epidemiologically equivalent, and evidence from the latter settings is interpreted according to its source and strength rather than as proof of onboard acquisition or transmission. The review further discusses prevention strategies and future perspectives from a One Health approach, including implications for travel medicine, maritime biosecurity, and preparedness for emerging infectious threats.

## 2. Methods

This narrative review was based on a structured search of the scientific literature relevant to animal reservoirs, zoonotic viruses, cruise ships, maritime environments, travel medicine, and One Health. We used the bibliographic databases PubMed/MEDLINE (NLM, NCBI, NIH), Scopus (Elsevier), and Web of Science (Clarivate) to identify peer-reviewed publications. We complemented searches with Google Scholar and relevant documents from international and national public health organizations, particularly the World Health Organization (WHO) (Geneva, Switzerland) and the U.S. Centers for Disease Control and Prevention (CDC) (Atlanta, GA, USA).

We used search terms individually and in combinations. They included “hantavirus”, “Andes virus”, “Seoul virus”, “zoonotic viruses”, “animal reservoirs”, “rodents”, “bats”, “migratory birds”, “wildlife”, “non-human primates”, “cruise ships”, “maritime tourism”, “ships”, “seaports”, “travel medicine”, “biosecurity”, “surveillance”, and “One Health”. We applied Boolean operators (AND/OR) as appropriate to combine pathogen-, reservoir-, and maritime-related concepts. We conducted the literature search up to 1 July 2026. We imposed no lower publication-date restriction; therefore, the search covered the available literature through 1 July 2026.

We screened titles and abstracts for relevance to the review objectives and examined the reference lists of selected publications to identify additional pertinent sources. We considered publications for inclusion if they addressed animal reservoirs or vectors of zoonotic viruses, zoonotic transmission in maritime or port environments, cruise-associated infectious risks, wildlife or environmental exposures during maritime tourism, travel medicine, biosecurity, outbreak preparedness, or One Health surveillance and prevention. We considered original studies, epidemiological and outbreak investigations, surveillance reports, reviews, guidelines, and authoritative public health reports. We excluded publications not substantively relevant to these themes.

Because cruise-specific evidence is limited, we also considered evidence from terrestrial, port, coastal, and wildlife-tourism settings when directly relevant to exposures along cruise itineraries; we interpreted this evidence as contextual or extrapolated, not as demonstrating onboard acquisition or transmission unless explicitly documented. For interpretive purposes, we distinguished three evidence categories throughout the review: (i) direct cruise-specific evidence, including documented events occurring aboard or epidemiologically linked to cruise travel; (ii) maritime-context evidence derived from seaports, commercial vessels, cargo terminals, provisioning areas, or other shipping-related environments; and (iii) destination-associated or contextual evidence derived from terrestrial, coastal, wildlife-tourism, or ecotourism settings. We considered only the first category as direct evidence of cruise-associated events while using the second and third categories to assess plausible exposure pathways and One Health relevance, without interpreting them as evidence of onboard acquisition or transmission.

We synthesized the selected literature narratively around the review’s principal themes: reservoir ecology, epidemiological risks associated with cruise travel, travel medicine and risk assessment, prevention and biosecurity, and One Health preparedness. We paid particular attention to the 2026 M/V Hondius Andes virus outbreak because of its direct relevance to zoonotic transmission and outbreak management in an expedition cruise setting. We did not perform a meta-analysis, formal risk-of-bias assessment, or systematic evidence grading, as these were beyond the scope of this narrative review.

## 3. Ecology and Diversity of Animal Reservoirs of Orthohantavirus and Other Zoonotic Viruses Relevant to Cruise Ships

The ecology of animal reservoirs plays a fundamental role in the emergence and re-emergence of zoonotic viruses, as reservoir distribution, population dynamics, and host–pathogen interactions strongly influence spillover risk in environments characterized by intense human mobility, including maritime tourism and international travel [21,22,23]. Environmental changes, the expansion of human mobility, and the growing interaction between natural ecosystems and tourism activities have favored complex scenarios of viral transmission in coastal regions, ports, and highly visited islands [24]. In this context, multiple animal species act as natural reservoirs or amplifiers of emerging viruses, facilitating the circulation of infectious agents with potential impact on passengers, crews, and port communities [25].

In this review, relevance to cruise travel encompasses the entire travel continuum, including pre-embarkation travel, port calls, shore excursions, and onboard activities. For several wildlife- and vector-associated viruses discussed below, the available evidence derives predominantly from terrestrial, coastal, or port environments; therefore, their inclusion reflects plausible exposure opportunities associated with cruise itineraries rather than documented transmission aboard cruise ships.

Wild and peri-urban rodents are the principal reservoirs of several medically important orthohantaviruses of the genus Orthohantavirus, family Hantaviridae [26]. These include Andes virus and Seoul virus, which are associated with specific rodent hosts and can cause severe disease in humans. Each species of orthohantavirus is usually associated with specific hosts, mainly rodents of the families Muridae and Cricetidae [8]. In Asia and Europe, viruses related to hemorrhagic fever with renal syndrome predominate, while in the Americas, orthohantaviruses associated with orthohantavirus cardiopulmonary syndrome stand out [7].

The etiopathogenesis of human orthohantavirus disease is characterized primarily by infection of vascular endothelial cells and a dysregulated host immune response that increases vascular permeability without extensive direct endothelial cytolysis. In hemorrhagic fever with renal syndrome, including disease caused by Seoul virus, endothelial dysfunction and capillary leakage predominantly affect the renal microcirculation, contributing to thrombocytopenia, hemorrhagic manifestations, and acute kidney injury. In orthohantavirus cardiopulmonary syndrome, including severe Andes virus infection, pulmonary capillary leakage may result in non-cardiogenic pulmonary edema, hypoxemia, myocardial dysfunction, and shock. Andes virus is also distinctive among orthohantaviruses because it can be transmitted person-to-person [7,26].

From an epidemiological perspective, Seoul virus and Andes virus should be clearly distinguished. Seoul virus is maintained predominantly in *Rattus* spp., has a broad geographic distribution associated with commensal rats, typically causes hemorrhagic fever with renal syndrome, and is acquired mainly through exposure to contaminated rodent excreta; person-to-person transmission has not been established. Its relevance to cruise travel therefore lies primarily in seaports, warehouses, provisioning areas, commercial vessels, and other maritime infrastructure where *Rattus* spp. may occur. Andes virus, in contrast, circulates in South America in wild sigmodontine rodent reservoirs, is primarily associated with orthohantavirus cardiopulmonary syndrome, and can undergo person-to-person transmission. In cruise travel, its principal relevance is therefore terrestrial exposure before embarkation or during shore activities, with the possibility of subsequent onboard human-to-human transmission after introduction, as illustrated by the M/V Hondius outbreak [7,10,26,27,28,29,30,31,32].

Of particular importance is the Seoul virus due to its association with urban rats of the genus *Rattus*, especially *Rattus norvegicus* and *Rattus rattus*, widely distributed in seaports, cargo terminals, warehouses, and commercial vessels [33]. These species have a remarkable capacity to adapt to urban and maritime environments, favoring viral persistence and international dispersal through trade routes and maritime transport [34,35]. However, the evidence supporting Seoul virus circulation in maritime environments derives predominantly from seaports, cargo facilities, urban settings, and commercial vessels rather than from cruise ships specifically. Thus, its relevance to cruise ships should currently be regarded primarily as an extrapolation from the well-established ecology of *Rattus* spp. in port and commercial maritime environments and their potential ability to access passenger vessels [4,36].

The remarkable adaptability of these rodents to ships, cargo terminals, food storage facilities, and urban coastal environments facilitates long-term viral maintenance in maritime ecosystems [37]. In addition, rodent infestations associated with waste accumulation and port infrastructure deterioration may increase opportunities for indirect human exposure to contaminated excreta [38].

Cruise ships and tourist ports offer favorable conditions for the proliferation of rodents due to the availability of food, organic waste, and closed structures that facilitate shelter and reproduction [39,40]. Although cases of orthohantavirus directly associated with cruise ships are rare, the persistent presence of infected reservoirs in port areas poses a potential risk of exposure, particularly during logistics operations, food storage, and visits to destinations with high rodent infestation [35]. Likewise, disorderly urban sprawl and health deterioration in some coastal regions have increased the interaction between humans and peri-urban reservoirs [41,42].

In addition to rodents, other mammals and vertebrates play an important role in the epidemiology of emerging viral zoonoses linked to maritime tourism [14]. Bats have become especially important as reservoirs of various RNA viruses, including coronaviruses, filoviruses, paramyxoviruses, and lyssaviruses [43]. Their extraordinary biological diversity, wide geographical distribution, and flight capacity facilitate pathogen dispersal across multiple island and coastal ecosystems [44]. In tropical tourist destinations and ecotourism excursions, indirect contact with caves, forests, and bat habitats can increase the risk of viral exposure [45].

The growing interest in ecotourism activities in caves, nature reserves, and jungle areas visited during cruise itineraries has expanded human–wildlife interfaces. Migratory birds also represent reservoirs and dispersal vehicles for important zoonotic viruses, especially avian influenza viruses [46]. Migratory routes connect continents and marine ecosystems, allowing the introduction of viral strains into ports, wetlands, and coastal areas frequented by tourists [47]. Outbreaks of highly pathogenic avian influenza have demonstrated the ability of these viruses to spread globally through migratory movements, trade, and human activities [48]. In maritime scenarios, the interaction between wild birds, port facilities, and coastal urban environments favors opportunities for interspecific transmission [49,50].

On the other hand, non-human primates and other wild mammals play an important role in the ecology of arboviruses such as yellow fever, particularly in jungle regions of South America and Africa [51,52]. Cruises that include excursions to tropical forests, natural parks, or ecological reserves can increase the risk of exposure to mosquito vectors and relevant wildlife hosts [53]. Similarly, other emerging zoonotic viruses, including orthopoxviruses and emerging arboviruses, have been related to wild mammals in ecosystems altered by human and tourist activities [54,55]. For rabies/lyssaviruses and reservoir-associated orthopoxviruses, relevance to cruise travel is likewise primarily destination-associated, reflecting potential wildlife exposure during shore excursions or ecotourism activities rather than documented animal-to-human transmission aboard cruise ships [56].

The geographical distribution of these reservoirs reflects their ecological and biogeographical constraints; however, global connectivity through international maritime transport and cruise tourism may facilitate the movement of infected hosts and vectors, as well as the introduction of synanthropic reservoir species in port environments, thereby linking distant endemic regions through anthropogenic mobility networks [57,58]. Tropical islands are particularly vulnerable ecosystems, where the introduction of invasive species, such as rodents, can profoundly alter ecological dynamics and facilitate the transmission of zoonotic pathogens [59]. In addition, the increase in tourism in polar and subpolar regions has raised concerns about the introduction of infectious agents into previously isolated ecosystems [60,61].

Climate change is the main factor modifying the dynamics of zoonotic virus transmission in maritime and coastal environments [62]. Global temperatures, altered precipitation patterns, and more frequent extreme weather events directly influence reservoir and vector distributions [63]. These changes favor the geographic expansion of arbovirus-transmitting mosquitoes, alter rodent reproductive cycles, and modify wild-bird migration [64,65,66]. Climate phenomena such as the El Niño–Southern Oscillation have been associated with rodent population increases and orthohantavirus outbreaks in different regions [4,67]. At the same time, deforestation, coastal urbanization, and biodiversity loss create ecological imbalances that increase opportunities for contact between humans and infected wildlife [68].

From a One Health perspective, maritime and coastal scenarios are dynamic interfaces where human, animal, and environmental factors converge to favor the emergence of viral zoonoses [69]. Ports, cruise terminals, and tourist destinations serve as nodes of ecological and epidemiological interaction, where global mobility facilitates the introduction and spread of pathogens between continents [70,71].

In these contexts, integrated surveillance should incorporate animal reservoir monitoring, vector control, environmental surveillance, and early detection systems in travelers and crews [72]. Likewise, cooperation among health authorities, environmental sectors, the shipping industry, and travel medicine specialists is essential to strengthen preparedness against future zoonotic threats associated with international maritime tourism [73,74]. Table 1 summarizes the main animal reservoirs, transmission pathways, and ecological characteristics of zoonotic viruses relevant to maritime tourism.

## 4. Epidemiological Risks of Viral Zoonoses on International Cruises

International cruises represent particularly complex scenarios for the transmission of infectious diseases due to the simultaneous interaction of epidemiological, environmental, and social factors [112]. The combination of high population density, accelerated global mobility, and contact with multiple ecosystems makes these vessels favorable spaces for the introduction, amplification, and dispersion of emerging zoonotic viruses [20]. Although historically health care on cruise ships has focused on gastrointestinal and respiratory diseases, in recent years, there has been increased concern about viral zoonoses associated with animal reservoirs and vectors present in tropical tourist destinations and international ports [113].

For conceptual clarity, infections associated with cruise travel should not necessarily be interpreted as infections acquired onboard. Five epidemiological scenarios should be distinguished: (i) infections acquired onboard through exposure to infected persons, vectors, or reservoir hosts; (ii) infections introduced onboard by passengers or crew who acquired infection before embarkation; (iii) infections acquired in port environments; (iv) infections acquired during shore excursions or ecotourism activities; and (v) the potential transport of vectors or reservoir hosts by vessels between ports and geographic regions. For many of the zoonotic viruses discussed in this review, particularly arboviruses and wildlife-associated infections, available evidence relates primarily to port, coastal, terrestrial, or ecotourism exposures rather than documented onboard transmission [36,114]. Accordingly, the evidence discussed below is interpreted according to its source setting, with documented cruise-specific events distinguished from maritime-context evidence and from broader destination-associated or ecological extrapolation.

One of the main factors that facilitates the transmission of infectious diseases on cruise ships is the inherent overcrowding of this mode of transport, as thousands of passengers and crew share relatively confined spaces for extended periods, including restaurants, theaters, swimming pools, gyms, and recreational areas [115]. These conditions facilitate close contact and the transmission of infectious agents, especially when there is high turnover among travelers from different continents [5]. The global experience during the COVID-19 pandemic evidenced the epidemiological vulnerability of cruise ships as environments that amplify infectious outbreaks [94]. However, beyond respiratory viruses, these conditions can also favor the late detection and spread of viral zoonoses acquired during port calls or tourist excursions [70].

Viral zoonoses of relevance to maritime travelers include orthohantaviruses, mainly associated with wild and peri-urban rodents in ports and coastal destinations [74]. Direct evidence of orthohantavirus acquisition from rodent reservoirs aboard cruise ships remains extremely limited [116]. For Seoul virus in particular, available evidence derives mainly from infected *Rattus* spp. in seaports, cargo facilities, urban environments, and commercial vessels; therefore, the proposed risk to cruise ships represents an epidemiologically plausible extrapolation from these maritime settings rather than a well-documented cruise-specific transmission pattern [117]. Potential exposure may nevertheless occur if infected rodents gain access to vessels or during port, provisioning, storage, or shore-based activities involving rodent-contaminated environments [29,118].

The unprecedented 2026 outbreak aboard the expedition cruise ship M/V Hondius represents the first documented cruise-ship-associated cluster of ANDV infection. The vessel departed Ushuaia, Argentina, on 1 April 2026 and subsequently travelled through Antarctica and several South Atlantic islands. A total of 13 cases were identified, including 12 laboratory-confirmed and one probable case, with three deaths, corresponding to a case-fatality ratio of 23% (https://www.who.int/emergencies/disease-outbreak-news/item/2026-DON611) (accessed on 12 September 2026) [119]. The precise zoonotic source of the outbreak was not established. WHO considered acquisition on land before embarkation the most likely explanation for the initial infection(s), but emphasized that the exact source and route of exposure remained undetermined. In contrast, epidemiological and genomic investigations supported subsequent limited human-to-human transmission aboard the vessel (https://www.who.int/emergencies/disease-outbreak-news/item/2026-DON611) (accessed on 12 September 2026) [119]. Importantly, no evidence demonstrated zoonotic transmission from an infected animal reservoir onboard or the existence of an established infected rodent population on the vessel. The outbreak therefore illustrates how a zoonotic infection probably acquired before embarkation may subsequently be introduced and amplified within a confined maritime environment. The international response included medical evacuation, laboratory coordination, repatriation, and contact tracing across 33 countries and territories. All identified contacts completed follow-up without detection of additional secondary cases, and WHO declared the outbreak contained on 2 July 2026 (https://www.who.int/emergencies/disease-outbreak-news/item/2026-DON611) (accessed on 12 September 2026) [74,120].

Meanwhile, mosquito-borne arboviruses represent important travel-associated risks for cruise ship passengers and crews. However, exposure generally occurs at ports of call, coastal destinations, or during shore excursions rather than onboard. Dengue remains one of the main infectious disease risks in tropical regions visited by maritime tourism, particularly in the Caribbean, Latin America, and Southeast Asia [121]. The geographic expansion of *Aedes aegypti* and *Aedes albopictus*, driven by climate change and urbanization, has increased dengue incidence in many tourist destinations. Similarly, chikungunya and Zika virus disease have caused major epidemics in areas frequently visited by international cruises, affecting residents and travelers [122,123]. Thus, their relevance to cruise medicine derives principally from itinerary-associated exposure and the potential for infected travelers to become symptomatic during or after the voyage, creating challenges for diagnosis and maritime health surveillance.

Yellow fever remains a significant concern in certain regions of Africa and South America [87]. Excursions to endemic jungle areas can expose unvaccinated passengers to infected mosquitoes and jungle reservoirs, especially non-human primates [88,89,124]. Likewise, the ongoing emergence and re-emergence of arboviruses pose new challenges for travel medicine and biosecurity on cruise ships. Accordingly, the relevance of dengue, chikungunya, Zika, and yellow fever to cruise travel should be interpreted primarily in terms of destination- and itinerary-associated mosquito exposure. Onboard transmission is not considered the predominant epidemiological scenario for these arboviruses, although vector introduction onto vessels remains a potential biosecurity concern. Other zoonotic viruses of interest include avian influenza associated with migratory birds and coastal environments, emerging orthopoxviruses, and respiratory viruses with pandemic potential [125].

Historical evidence shows that shipping and international tourism have contributed to the global spread of infectious diseases. Various outbreaks of influenza, coronavirus, and vector-borne diseases have been linked to maritime travel and international mobility [126,127,128,129,130]. During the COVID-19 pandemic, multiple cruise ships experienced extensive outbreaks, highlighting the limitations of onboard health surveillance and response systems [115,131,132]. Although most events reported on cruise ships involve respiratory and gastrointestinal infections, the risk of introducing emerging viral zoonoses remains a growing concern due to increased ecotourism and the expansion of routes into tropical and ecologically sensitive regions.

## 5. Travel Medicine and Risk Assessment in Passengers and Crew

The growing expansion of international maritime tourism has made cruise ships relevant scenarios for travel medicine and the prevention of emerging infectious diseases [133]. Accelerated mobility between continents, contact with multiple ecosystems, and constant interaction between passengers and crews of different origins create conditions that favor exposure to infectious agents, including various zoonotic viruses [134,135]. In this context, comprehensive risk assessment before, during, and after the voyage is an essential component to reduce the impact of emerging diseases on passengers, maritime workers, and host communities [136].

The risk factors associated with viral zoonoses on cruise ships are multiple and depend on both individual and occupational characteristics. Individual factors include advanced age, chronic diseases, immunosuppression, pregnancy, and pre-existing cardiovascular or respiratory conditions, which can increase susceptibility to serious infections and clinical complications [137,138]. This is especially relevant given that a significant proportion of cruise passengers are older adults, a group particularly vulnerable to emerging infectious diseases. Likewise, the lack of prior vaccination, ignorance of epidemiological risks, and participation in high-exposure ecotourism activities increase the probability of acquiring zoonotic infections during the trip [139].

From an occupational perspective, crew members spend prolonged periods aboard vessels and may repeatedly interact with operational environments throughout successive itineraries. Certain duties, including food handling, cleaning, maintenance, storage, and waste management, may involve contact with food-storage areas, waste streams, pest-prone spaces, or environmentally contaminated areas relevant to shipboard biosecurity [36,40,140,141]. However, cruise-specific epidemiological evidence demonstrating a higher incidence of zoonotic infections among these occupational groups remains limited; these activities should therefore be considered potential exposure pathways rather than established occupational risk factors [142]. Crew populations may also be affected by person-to-person transmission of communicable infections onboard, as demonstrated during outbreaks on maritime vessels [143,144].

Pre-trip evaluation is one of the fundamental pillars of travel medicine, especially for passengers who will make itineraries to tropical regions or areas with active circulation of viral zoonoses [145]. This assessment should carefully consider the entire cruise itinerary, port calls, planned activities, and the epidemiological situation of the destinations visited. It is important to identify risks associated with exposure to mosquitoes, contact with wildlife, visits to rural or jungle areas, and participation in ecotourism excursions [146]. Relevant medical history, immune status, and possible contraindications to vaccines or prophylactic medications should also be evaluated [147].

Pre-trip recommendations should include detailed education on preventative measures and safe behaviors during excursions [148]. Travelers should receive information about the risk of mosquito-borne diseases, including dengue, chikungunya, and Zika virus disease, particularly in tropical regions of the Caribbean, Latin America, Africa, and Southeast Asia [149,150]. Similarly, there may be a risk of exposure to yellow fever on certain itineraries, especially during excursions to endemic jungle areas; the recommendations must be individually adapted according to the traveler’s profile and the epidemiological characteristics of each sea route [151,152].

Vaccination represents one of the most effective preventive strategies for maritime travelers. Depending on the itinerary, destination-specific epidemiology, and individual traveler characteristics, routine, recommended, and required vaccinations should be reviewed before cruise travel, including influenza and COVID-19 and, when indicated, yellow fever, hepatitis A, hepatitis B, and other travel-related vaccines [112]. In some cases, international yellow fever vaccination certificates remain mandatory requirements for entry into certain countries [153]. In addition to vaccines, personal protective measures are essential to reduce exposure to arthropod vectors and animal reservoir hosts. These include using repellents, wearing long-sleeved clothing, using mosquito nets, protecting against arthropod bites, and avoiding contact with wild animals or environments that rodent droppings may contaminate [154]. Also, proper hand hygiene and food safety practices should be promoted during travel.

Before embarkation, vaccination status should be reviewed based on the complete itinerary, including routine vaccines, destination-specific recommendations, and vaccines required for entry into particular countries. When applicable, documentation of mandatory vaccination should be verified, particularly the International Certificate of Vaccination or Prophylaxis for yellow fever, before travel. Routine measurement of vaccine-specific antibody titers in all cruise passengers is not generally indicated; serological testing may be considered selectively when previous vaccination records are unavailable or unreliable, when confirmation of immunity is clinically relevant, or in specific circumstances supported by current travel-medicine guidelines [155,156].

The clinical manifestations of viral zoonoses in maritime travelers are often non-specific and represent a significant diagnostic challenge [36,157]. Many emerging infections begin with acute febrile symptoms, headache, myalgias, and malaise, making it difficult to differentiate between various viral etiologies clinically [158]. Orthohantaviruses, for example, may initially manifest with influenza-like symptoms before progressing to severe cardiopulmonary or renal involvement [6,58]. Similarly, dengue, chikungunya, and Zika share early clinical manifestations that make differential diagnosis difficult, especially in resource-limited settings or during extended travel [159].

Diagnostic challenges increase because cruise ships are itinerant and incubation periods can be prolonged. Some passengers may develop symptoms after leaving the ship or after returning to their home countries, making it difficult to identify the source of exposure; likewise, the coexistence of multiple endemic diseases in certain tropical destinations increases the diagnostic complexity [128,160]. Arbovirus co-infection, the simultaneous circulation of respiratory viruses, and diagnostic limitations at sea represent additional obstacles to timely case recognition [161].

In this context, clinical and laboratory surveillance is of critical importance both in seaports and on board ships. Cruise ship medical services should have clear protocols in place for the early identification, isolation, and initial management of suspected cases of emerging infectious diseases [70]. Syndromic surveillance can facilitate the early detection of unusual patterns of disease among passengers and crew members, allowing control measures to be activated before outbreaks spread [162]. In addition, access to rapid diagnostic tests, molecular tools, and telemedicine systems can significantly improve healthcare response capacity in maritime environments [36].

Collaboration between port authorities, national health systems, reference laboratories, and the shipping industry is essential to strengthen international epidemiological surveillance [163]. From a One Health perspective, travel medicine must integrate human, environmental, and veterinary epidemiological information to anticipate emerging risks associated with maritime tourism [164]. Adequate preparedness, health education, and continuous surveillance represent fundamental tools to reduce the impact of viral zoonoses on international cruises and strengthen global health security in the face of future emerging infectious threats [165].

## 6. Prevention, Biosecurity and Infection Control on Cruise Ships

The prevention and control of infectious diseases on international cruise ships are priority challenges for global public health due to high human mobility, population concentration, and the constant interaction among passengers, crew, and various port ecosystems [70]. Cruise ships function as dynamic environments where environmental, biological, and operational factors converge, favoring the introduction and spread of infectious agents, including emerging zoonotic viruses [137]. In this context, biosecurity strategies must integrate epidemiological surveillance programs, reservoir and vector control, environmental sanitation, health education, and international cooperation under a comprehensive One Health approach [18].

One fundamental pillar of preventing viral zoonoses on cruise ships is rodent and vector control programs both on ships and in port terminals [39,166]. Rodents represent important reservoirs of various zoonotic pathogens, including orthohantaviruses, leptospires, and other infectious agents with potential health impacts; in particular, urban species such as *Rattus norvegicus* and *Rattus rattus* have a great capacity to adapt to maritime and port environments, favoring the colonization of warehouses, kitchens, and food storage systems [140,167,168,169]. The presence of these animals on ships and in ports can facilitate the contamination of surfaces and food with infected urine, feces, and secretions [170].

Effective rodent control programs should include regular inspections, ongoing monitoring, sealing of entry points, and proper handling of food and waste [40,170]. Integrated surveillance, trapping, and pest management programs are essential to prevent infestations and the establishment of invasive vectors at ports of entry; these measures are particularly important in tropical cruise ports, where *Aedes* mosquitoes can proliferate and increase the risk of dengue, chikungunya, and Zika virus transmission among travelers and local populations [141]. Prevention should therefore distinguish between destination-associated and onboard risks. For passengers, the principal preventive measures against mosquito-borne arboviruses include destination-specific risk assessment, vaccination when applicable, repellents, protective clothing, and mosquito-bite avoidance during port visits and shore excursions. Onboard measures should focus primarily on preventing the introduction and establishment of mosquito vectors through surveillance, elimination of breeding sites, and appropriate vector-control procedures [171,172].

At ports located in areas with intense arboviral transmission, the potential role of targeted disinsection or spatial vector-control measures could also be evaluated, particularly when entomological surveillance indicates substantial vector activity. However, established travel-medicine or vector-control guidance does not currently support routine compulsory misting of passengers with insect repellents during disembarkation. Instead, personal repellents should be applied to exposed skin or clothing according to product instructions, and any port- or vessel-based insecticide intervention should be directed primarily at vector control, evaluating efficacy, safety, insecticide resistance, environmental impact, and regulatory acceptability [173,174].

Food safety is another priority aspect within the preventive strategies on international cruises [175]. Contaminated food can transmit infectious agents, including viruses, bacteria, and parasites. Kitchens and food preparation areas require strict hygiene measures, temperature control, sanitary supervision, and continuous training of handling personnel [176]. Likewise, food supply at international ports involves risks of microbiological contamination and exposure to animal reservoirs during transport and storage. Additionally, regular health inspections and the implementation of hazard analysis systems and critical control points are essential to ensure safe feeding conditions on board [177,178].

Early detection and rapid response to suspected cases are critical components of infection control on cruise ships. The experience gained during the COVID-19 pandemic evidenced the importance of having clear protocols for the identification, isolation, and initial management of emerging infectious diseases in maritime environments [132,142]. Medical services on board should have syndromic surveillance systems capable of early detection of symptoms compatible with communicable infectious diseases [179]. Early identification of fever, respiratory symptoms, rashes, or bleeding syndromes allows isolation and epidemiological control measures to be activated quickly [180].

Response protocols should include initial clinical evaluation, immediate notification to health authorities, contact tracing, and coordination with destination ports for hospital referral when necessary [181]. It is also important to have adequate isolation areas, personal protective equipment, and access to basic diagnostic tools [182]. Telemedicine and digital communication technologies have become increasingly relevant for supporting clinical decision-making at sea, particularly during complex or emerging infectious events [183,184].

Health education for passengers is a critical preventive tool to reduce the risk of zoonotic diseases and other infections associated with maritime travel [128,185]. Travelers should receive clear information on personal hygiene measures, mosquito bite prevention, food safety, and safe behaviors during ecotourism excursions [146]. Such guidance could be reinforced through clear visual educational materials and itinerary-specific notifications delivered through cruise mobile applications or passengers’ personal devices before and during shore excursions, emphasizing appropriate behaviors according to the epidemiological and ecological risks of each destination. In addition, it is important to encourage early notification of symptoms compatible with infectious diseases and promote adherence to medical and health recommendations during the trip; therefore, adequate risk perception and effective communication are essential to strengthen passengers’ active participation in prevention strategies [116,136].

International regulations play an essential role in the surveillance and sanitary control of international cruise ships. The World Health Organization’s International Health Regulations establish regulatory frameworks for the prevention of the international spread of disease and strengthen coordination between countries and port authorities [186]. Similarly, programs such as the U.S. Centers for Disease Control and Prevention’s Vessel Sanitation Program have contributed significantly to improving sanitary conditions on cruise ships through periodic inspections and specific hygiene and environmental control standards [136].

Maritime health surveillance requires close cooperation between national authorities, international organizations, shipping companies, and public health services [186]. Seaports are strategic points for detecting emerging diseases and implementing preventive measures to avoid the cross-border introduction of pathogens [187]. From the One Health perspective, prevention and biosecurity strategies must integrate human, animal, and environmental components to strengthen global preparedness against future zoonotic threats associated with international maritime tourism [188].

These preventive, surveillance, and response measures should be implemented throughout the cruise travel continuum, from itinerary planning and pre-embarkation assessment to onboard operations, shore excursions, outbreak management, and post-disembarkation follow-up, as summarized in Table 2.

## 7. One Health Approach, Research Gaps, and Future Perspectives for the Prevention of Viral Zoonoses in Maritime Tourism

The particular value of a One Health approach in maritime tourism lies in linking human, animal, vector, and environmental information across the cruise-travel continuum. However, a major knowledge gap is the scarcity of cruise-specific evidence that can distinguish onboard acquisition, infections introduced by passengers or crew, port-associated exposure, shore-excursion exposure, and the transport of vectors or reservoir hosts by vessels. Future surveillance and research should therefore apply standardized exposure classifications and link passenger and crew illness with itineraries, port calls, excursion histories, and relevant animal, vector, and environmental findings [3,189,190,191]. Such integration is particularly important in maritime tourism because vessels connect multiple ecological settings and health jurisdictions within short periods.

Cruise travel creates a distinctive epidemiological interface linking vessels, ports, passengers, crew members, vectors, wildlife, and diverse coastal and terrestrial ecosystems. A maritime One Health surveillance model should therefore connect onboard syndromic surveillance with itinerary-specific information from ports and destinations, including animal reservoir activity, vector abundance, environmental conditions, and relevant ecological changes [136,186,192]. Particular attention should be given to cruise terminals, provisioning areas, high-biodiversity destinations, and locations where vessels may facilitate the introduction of invasive rodents or vectors. Coordinated monitoring of rodents, mosquitoes, birds, and other relevant animals should be linked, when feasible, with human surveillance rather than conducted as an independent activity [186,193,194,195,196].

Recent advances in molecular diagnostics, genomics, and metagenomics offer important opportunities for maritime One Health surveillance [197,198]. Applied to cruise travel, these technologies could support rapid characterization of unusual onboard clusters, comparison of pathogens detected in passengers with those identified in potential animal reservoirs or environmental samples, and differentiation between imported infections and possible onboard transmission [199]. Environmental surveillance of wastewater and selected high-risk surfaces aboard cruise ships, combined with portable molecular diagnostic tools and genomic sequencing when available, may further strengthen early detection and outbreak investigation [200,201]. However, their feasibility, diagnostic yield, cost-effectiveness, and operational value for routine cruise-ship surveillance require further evaluation.

Artificial intelligence (AI)-supported information technologies may further strengthen integrated One Health surveillance by combining heterogeneous data streams, including human syndromic and laboratory data, genomic information, wastewater signals, animal-reservoir and vector surveillance, meteorological and satellite-derived environmental variables, travel itineraries, and human mobility data. Machine-learning, deep-learning, natural-language-processing, and geospatial AI approaches can identify complex or nonlinear epidemiological patterns, detect unusual signals, and generate early-warning forecasts of changes in infectious-disease risk. In maritime tourism, these tools could support dynamic risk assessment of ports and itineraries and help prioritize surveillance and preventive interventions. Realizing this potential will require internationally interoperable and ethically governed data architectures that facilitate timely cross-sector and cross-country access to appropriately de-identified or aggregated human health data while ensuring robust protection of individual privacy. Privacy-preserving AI approaches, including federated and distributed analytic models, could enable information from different jurisdictions and sectors to be analyzed jointly without requiring unrestricted transfer or centralization of identifiable personal data. Such frameworks could substantially strengthen the integration of human, animal, vector, environmental, genomic, mobility, and maritime surveillance data while maintaining requirements for transparency, explainability, external validation, data governance, and protection against algorithmic bias [202,203].

In this regard, cruise itineraries routinely involve multiple countries and health jurisdictions, making interoperable surveillance and rapid information-sharing particularly important [58]. Operational One Health preparedness should establish communication mechanisms among ship medical services, cruise operators, port health authorities, reference laboratories, veterinary and environmental agencies, and national public health authorities. Standardized procedures for specimen referral, exposure classification, contact follow-up, and the exchange of epidemiological and genomic information would facilitate investigations of events involving multiple jurisdictions. The COVID-19 pandemic demonstrated both cruise ships’ vulnerability to infectious disease outbreaks and the importance of international collaboration in implementing effective control measures [115,162].

Future One Health challenges for maritime tourism include climate change, changing vector and reservoir distributions, expansion of cruise routes into ecologically sensitive regions, and increasing participation in wildlife-based and ecotourism activities. Changes in temperature, precipitation patterns, and ecosystems may alter the distribution of vectors and reservoirs, increasing opportunities for zoonotic exposure [204]. In addition, growing ecotourism activities may increase human exposure to wildlife and natural habitats [205,206,207].

Important research priorities include prospective studies quantifying zoonotic infections among cruise travelers according to the most probable exposure setting; studies evaluating whether infected vectors or reservoir hosts are transported aboard passenger vessels; assessment of wastewater, environmental, and vector surveillance specifically for zoonotic pathogens in cruise settings; and genomic epidemiological studies capable of distinguishing imported infections from true onboard transmission. Further research should also evaluate the effectiveness and feasibility of integrated surveillance models linking shipboard clinical data with port, animal, vector, and environmental information. Addressing these gaps through multidisciplinary and international research will be essential for translating the One Health concept into operational preparedness for safer and more resilient maritime tourism [208,209,210].

## 8. Limitations

This review was designed as a structured narrative review intended to integrate heterogeneous evidence spanning animal-reservoir ecology, travel medicine, maritime health, outbreak investigations, surveillance, and One Health, rather than to provide an exhaustive systematic synthesis or quantitative estimate of effect. To strengthen methodological transparency, we applied a structured literature search across multiple bibliographic databases and authoritative public-health sources, predefined thematic search terms and eligibility criteria, a prespecified search cutoff, and an explicit distinction between cruise-specific evidence and contextual evidence extrapolated from port, terrestrial, coastal, and wildlife-tourism settings. Nevertheless, as with narrative evidence synthesis, source selection and interpretation remain potentially susceptible to publication and selection bias. Second, cruise-specific evidence on zoonotic viruses and animal reservoirs remains scarce, heterogeneous, and largely based on case reports, outbreak investigations, surveillance reports, and extrapolation from ports or terrestrial tourism settings. Accordingly, associations described as relevant to cruise travel should not be interpreted as evidence of onboard acquisition or transmission unless specifically demonstrated. Third, underdiagnosis and inconsistent reporting may underestimate the true burden. Finally, evidence concerning the M/V Hondius outbreak and other emerging events continues to evolve, limiting definitive conclusions regarding exposure sources, transmission pathways, and the effectiveness of specific preventive measures.

## 9. Conclusions

Maritime tourism and the global expansion of international cruise travel have generated increasingly complex epidemiological interfaces involving vessels, ports, coastal ecosystems, tourist destinations, and wildlife. Zoonotic infections identified among cruise travelers may originate at different points along this continuum, including before embarkation, during port calls or shore excursions, and, for some pathogens, onboard the vessel. Cruise-associated infection should therefore not be considered synonymous with onboard acquisition. Rather, cruise itineraries can connect highly mobile populations with geographically diverse zoonotic hazards and may facilitate the detection, geographic movement, and, for pathogens capable of person-to-person transmission, subsequent amplification of infections acquired in different settings.

Peri-urban rodents, bats, migratory birds, non-human primates, and other wildlife species can play distinct ecological roles as reservoir, maintenance, or amplifying hosts in the transmission cycles of zoonotic viruses relevant to destinations visited by cruise travelers. However, much of the available evidence derives from terrestrial, port, coastal, and wildlife-tourism settings rather than documented onboard transmission. Climate change, accelerated urbanization, environmental alteration, and expanding ecotourism may further modify these exposure interfaces by changing the distribution of vectors and reservoir hosts.

Given the limited cruise-specific evidence, these pathogens should be regarded as potential or emerging public health concerns in the context of international maritime tourism rather than as uniformly established or increasing cruise-associated threats. Nevertheless, appropriate prevention and biosecurity strategies remain important for reducing potential risks to travelers, crew members, and port communities. Rodent and vector control programs, environmental sanitation, epidemiological surveillance, health education, and onboard clinical preparedness are fundamental pillars for strengthening maritime health security. In this context, the One Health approach emerges as an indispensable tool for integrating human, animal, and environmental surveillance in complex maritime scenarios.

Finally, strengthening international cooperation, molecular monitoring of emerging pathogens, and preparedness for future zoonoses will be decisive in ensuring safer, more resilient, and more sustainable maritime tourism amid the global epidemiological challenges of the 21st century.

## Figures and Tables

**Table 1:** Reservoirs, vectors, transmission pathways, and level of evidence for zoonotic viruses relevant to international cruise travel.

Viral Disease/Group	Natural Reservoir or Amplifying Host	Vector, If Applicable	Main Transmission Route to Humans	Evidence in Cruise Ships/Ports	Level of Cruise-Related Evidence	Relevance to Cruise Travel	References
Orthohantaviruses	Rodents; species-specific associations including *Rattus*, *Oligoryzomys*, and *Peromyscus*	None	Mainly inhalation of aerosolized rodent excreta; person-to-person transmission documented for Andes virus	Seoul virus and infected *Rattus* documented in seaports and maritime infrastructure; Andes virus cluster documented in cruise travel, but no infected onboard rodent reservoir demonstrated	Direct cruise evidence for Andes virus human transmission; maritime-context evidence for Seoul virus reservoir exposure	Port, provisioning, storage, and rodent-contaminated environments; possible onboard amplification after introduction of Andes virus	[10,27,28,29,30,31,32]
Dengue virus	Humans are the principal amplifying hosts in the urban cycle; non-human primates participate in sylvatic cycles in some settings	*Aedes aegypti*, *Aedes albopictus*	Mosquito bite; sexual and vertical transmission also occur, but less frequently and with less evidence than in Zika	Evidence primarily from endemic ports and tourist destinations rather than onboard transmission	Destination-associated evidence	Exposure mainly during port calls and shore activities in endemic areas	[75,76,77,78]
Chikungunya virus	Humans in urban transmission cycles; non-human primates and other vertebrates participate in sylvatic cycles	*Aedes* spp.	Mosquito bite	Primarily documented in endemic destinations rather than aboard cruise ships	Destination-associated evidence	Port and shore-excursion exposure	[79,80,81]
Zika virus	Humans are important amplification hosts; non-human primates participate in sylvatic cycles	*Aedes* spp.	Mosquito bite; sexual and vertical transmission also occur	Evidence primarily from endemic terrestrial destinations	Destination-associated evidence	Shore excursions and travel to endemic areas; reproductive-health implications	[81,82,83,84]
Yellow fever virus	Non-human primates are major amplifying hosts in sylvatic cycles	*Haemagogus*, *Sabethes*, and *Aedes* spp., according to transmission cycle	Mosquito bite	No established onboard reservoir-associated transmission; risk relates mainly to endemic destinations	Destination-associated evidence	Jungle and forest excursions in endemic regions	[85,86,87,88,89]
Avian influenza viruses	Wild aquatic and migratory birds; poultry may serve as amplifying hosts	None	Direct or environmental exposure to infected birds or contaminated materials	Relevant evidence derives mainly from coastal, port, poultry, and wildlife environments	Maritime-context/destination-associated evidence	Ports, coastal environments, and wildlife-associated excursions	[90,91,92]
SARS-related coronaviruses	Bats are important ancestral reservoirs; intermediate mammalian hosts may be involved	None	Primarily respiratory transmission after zoonotic introduction	Strong evidence for person-to-person outbreaks aboard cruise ships, but not for onboard animal-reservoir transmission	Direct cruise evidence for human transmission; contextual evidence for animal reservoirs	Introduction by infected travelers followed by possible onboard amplification	[93,94,95,96]
Nipah virus	*Pteropus* fruit bats; pigs may act as amplifying hosts in some outbreaks	None	Direct animal contact, contaminated food, or person-to-person transmission	No established cruise-specific transmission	Contextual/destination-associated evidence	Wildlife and food-related exposure during terrestrial travel or excursions	[97,98,99,100]
Rabies and lyssaviruses	Bats, dogs, and wild carnivores are important reservoir/maintenance hosts; cats are susceptible domestic hosts	None	Bite or saliva exposure	No established onboard reservoir-associated transmission	Destination-associated evidence	Domestic-animal and wildlife contact during port visits and excursions	[101,102,103,104,105,106]
Orthopoxviruses, including mpox-related viruses	Animal reservoir(s) remain incompletely defined; several rodents and other small mammals are implicated	Direct contact	Direct animal contact; human-to-human transmission may subsequently occur	No demonstrated onboard animal-reservoir transmission	Contextual/destination-associated evidence	Wildlife-tourism interfaces and exposure at destinations	[107,108,109,110,111]

**Note:** Evidence levels refer to the setting from which the available evidence derives: direct cruise-specific evidence, maritime-context evidence from ports or other shipping-related environments, and destination-associated/contextual evidence from terrestrial, coastal, wildlife-tourism, or ecotourism settings. These categories should not be interpreted as epidemiologically equivalent, and contextual evidence does not demonstrate onboard acquisition or transmission.

**Table 2:** One Health framework for preventing, detecting, and responding to zoonotic viral threats associated with international cruise travel (M = mandatory when required by applicable international, national, port-health, or entry regulations; R = recommended measure based on established guidance or good practice, but not universally mandatory; S = suggested or emerging measure proposed for evaluation or implementation in appropriate settings).

Phase or Operational Setting	Principal Risks and Considerations	Measures and Implementation Category	Primary Stakeholders
**Itinerary planning and pre-voyage risk assessment**	Travel to endemic regions, tropical ports, remote islands, forests, caves, wetlands, and other wildlife interfaces; limited medical and evacuation capacity in remote destinations.	[R] Assess the epidemiological and ecological situation at all destinations; identify areas with active zoonotic or vector-borne disease transmission; evaluate medical referral, evacuation, laboratory, and communication capacities; establish contingency plans before departure.	Cruise operators; itinerary planners; maritime medical teams; port authorities; travel-medicine specialists; public health agencies.
**Pre-embarkation assessment of passengers and crew**	Advanced age, pregnancy, immunosuppression, chronic cardiovascular or respiratory disease, incomplete vaccination, and recent exposure in endemic areas may increase the risk of infection or severe outcomes.	[M] Verify vaccination certificates when legally required; [R] review routine and destination-specific vaccinations and provide counseling; [S] consider selective serologic assessment when vaccination records are unavailable or unreliable.	Travel-medicine providers; healthcare professionals; cruise medical services; passengers and crew.
**Provisioning, loading, and port operations**	Introduction of rodents, mosquitoes, contaminated cargo, food, or other biological hazards through ports, warehouses, provisioning areas, and stored goods.	[R] Inspect cargo and provisioning areas; maintain food protection and waste controls; seal potential rodent entry points; implement trapping, pest-management, and vector-surveillance programs; [M] comply with vessel, port-health, sanitation, and vector-control requirements established by the competent authorities; [R] coordinate inspections between vessels and ports.	Cruise operators; port health authorities; pest-control personnel; food-safety services; veterinary and environmental authorities.
**Routine onboard prevention and environmental management**	Rodent infestation, mosquito breeding, contaminated surfaces or food, inadequate waste handling, and delayed recognition of febrile illness.	[R] Conduct scheduled inspections and environmental monitoring; ensure safe food storage and preparation; manage waste appropriately; eliminate vector-breeding sites; document pest sightings and corrective actions; [M] maintain sanitation, food-safety, and pest-control standards required by applicable maritime and public-health regulations.	Ship management; environmental health officers; food handlers; housekeeping and maintenance personnel; pest-control services.
**Shore excursions and ecotourism activities**	Exposure to rodent excreta, mosquitoes, bats, wild birds, non-human primates, and other wildlife in rural, jungle, cave, wetland, island, or peri-urban environments.	[R] Perform excursion-specific risk assessments; avoid entering visibly rodent-infested or poorly ventilated enclosed spaces; prevent direct contact with wildlife; use repellents and protective clothing; use qualified guides and mechanisms for recording relevant exposures.	Excursion operators; local guides; cruise staff; passengers; destination health and environmental authorities.
**Onboard syndromic surveillance**	Early manifestations of orthohantavirus and other zoonotic viral infections may be nonspecific and resemble influenza, COVID-19, or other acute febrile illnesses.	[R] Monitor fever, myalgia, headache, respiratory symptoms, rash, bleeding manifestations, and gastrointestinal symptoms; maintain accessible medical reporting systems; review cases for common itineraries, excursions, accommodations, or exposures; use telemedicine when necessary; [M] report events to the competent health authorities when notification is required under applicable regulations.	Ship physicians and nurses; epidemiological surveillance personnel; passengers and crew; shoreside medical support.
**Management of a suspected zoonotic viral infection**	Rapid clinical deterioration, uncertain transmission route, diagnostic limitations at sea, and possible exposure of close contacts.	[R] Isolate the patient when person-to-person transmission cannot be ruled out; provide supportive care and appropriate personal protective equipment; collect appropriate clinical specimens; [M] notify the captain, cruise operator, destination port, and competent health authorities when required by applicable regulations; [R] arrange laboratory testing and medical evacuation when indicated.	Ship medical team; cruise operator; port health authorities; receiving hospitals; reference laboratories.
**Investigation of a cluster or outbreak**	A common environmental exposure, multiple shore-based exposures, onboard transmission, or infection acquired before embarkation may produce similar temporal patterns.	[R] Develop a line list and exposure timeline; classify cases and contacts; conduct contact tracing; investigate cabins, food-storage areas, waste systems, excursions, and potential animal or vector exposures; [S] undertake molecular testing and genomic sequencing when available and epidemiologically appropriate.	National public health agencies; WHO and other international organizations; reference laboratories; cruise operators; veterinary and environmental investigators.
**Port arrival, evacuation, and cross-border coordination**	Multiple jurisdictions, notification delays, limited isolation capacity, and the international dispersal of passengers and crew may complicate containment efforts.	[R] Establish advance communication with the receiving port; coordinate safe disembarkation and medical referral; share standardized epidemiological and laboratory information; [M] apply applicable International Health Regulations and mandatory national or port-health notification procedures; [R] ensure continuity of care and contact follow-up across countries.	Port health authorities; national focal points; immigration and transport authorities; hospitals; cruise operators; international organizations.
**Post-disembarkation surveillance and follow-up**	Symptoms may develop after passengers or crew return home, obscuring the relationship with the cruise or a shared exposure.	[R] Provide written advice regarding incubation periods, warning symptoms, and where to seek care; retain passenger and crew contact information in accordance with applicable privacy requirements; [M] notify relevant national authorities when legally required; [R] monitor high-risk contacts for the pathogen-specific period and communicate relevant investigation findings internationally.	Cruise operators; passengers and crew; national surveillance systems; healthcare providers; public health authorities.
**Long-term One Health preparedness**	Climate change, urbanization, altered reservoir and vector distributions, expanding ecotourism, and incomplete integration of human, animal, and environmental surveillance.	[S] Integrate human case surveillance with rodent, mosquito, bird, and other wildlife monitoring, wastewater and surface surveillance, and environmental monitoring; strengthen portable diagnostics, genomics, interoperable data sharing, multidisciplinary training, simulation exercises, artificial intelligence-supported surveillance, and international research collaboration.	Public health, veterinary, wildlife, environmental, maritime, laboratory, and travel-medicine sectors; cruise industry; academic institutions.

WHO, World Health Organization. Measures should be tailored to the itinerary, local epidemiology, suspected pathogen, transmission pathway, traveler characteristics, and available maritime and port health resources. M, mandatory when required by applicable international, national, port-health, maritime, or entry regulations; R, recommended measure based on established guidance or good practice but not universally mandatory; S, suggested or emerging measure proposed for implementation or further evaluation. Classification may vary according to jurisdiction, itinerary, epidemiological context, and applicable regulations.

## Data Availability

No new data were created or analyzed in this study. Data sharing is not applicable to this article.
